# Comprehensive Stability Study of Vitamin D3 in Aqueous Solutions and Liquid Commercial Products

**DOI:** 10.3390/pharmaceutics13050617

**Published:** 2021-04-25

**Authors:** Žane Temova Rakuša, Mitja Pišlar, Albin Kristl, Robert Roškar

**Affiliations:** Faculty of Pharmacy, University of Ljubljana, Aškerčeva Cesta 7, 1000 Ljubljana, Slovenia; zane.temova.rakusa@ffa.uni-lj.si (Ž.T.R.); mitja.pislar@gmail.com (M.P.); albin.kristl@ffa.uni-lj.si (A.K.)

**Keywords:** antioxidants, cholecalciferol, degradation kinetics, food supplements, HPLC-UV, medicines, stability, stabilization

## Abstract

Vitamin D3 has numerous beneficial effects, such as musculoskeletal, immunomodulatory, and neuroprotective. However, its instability is the main obstacle to formulating quality products. Despite increased attention and growing use, data on vitamin D3 stability is scarce because data from individual studies is inconclusive and mostly qualitative. Therefore, we have systematically investigated the influence of various factors (temperature, light, oxygen, pH, concentration, and metal ions) on its stability in aqueous media using a stability-indicating HPLC-UV method. First-order kinetics fitted its degradation under all tested conditions except light and oxygen. In both cases, the established models in chemical kinetics were inappropriate and upgraded with the Weibull model. Metal ions and acidic conditions had the main destabilizing effect on vitamin D3 in aqueous media, but these solutions were successfully stabilized after the addition of ethylenediaminetetraacetic acid (EDTA), ascorbic acid, and citric acid, individually and in combination. EDTA showed the most significant stabilizing effect. Synergism among antioxidants was not observed. Our findings on vitamin D3 instability in aqueous media also correlated with its instability in commercial products. Vitamin D3 aqueous products require proper stabilization, thereby signifying the importance and contribution of the obtained results to the formulation of stable and quality products.

## 1. Introduction

The major role of vitamin D is to maintain calcium and bone homeostasis through its effects on the intestine, skeleton, parathyroid glands, and kidneys. Vitamin D promotes dietary calcium and phosphate absorption in the small intestine, mobilizes calcium from bones, and modulates the parathyroid function [1,2,3]. In recent years, many studies have paid attention to non-classic vitamin D actions, such as suppression of cell growth, regulation of apoptosis, modulation of immune responses, control of the nervous system, insulin secretion, and muscle function [4,5,6,7,8,9,10,11,12,13,14]. The number of such publications has substantially increased because of COVID-19, given the positive role of vitamin D on the immune response [15,16,17,18,19,20,21,22,23,24]. Vitamin D deficiency is a highly prevalent condition affecting approximately one billion people worldwide [25,26]. Pharmacologically, vitamin D is typically used in dosages of 400 to 1000 international units per day to prevent rickets in infants and young children, supplement the diet of pregnant and lactating women, and prevent and treat of primary and secondary osteoporosis [27]. Vitamin D3 can be found in various dosage forms: oil- or water-soluble capsules, tablets, concentrates (oil, powder, or water-dispersible form), injections, sprays, and oral solutions. Vitamin D3 medicines and food supplements, especially for paediatric use, are typically solutions with various excipients, including compounds that have a potential stabilizing effect, which was evaluated in this study. 

However, vitamin D3 is problematic in terms of stability, especially in aqueous media, which is evident from the shorter shelf lives of medicines and food supplements (one or two years) [27]. A study dealing with vitamin D3 stability in liquid prescription medicines and supplements after being opened showed that its content decreased considerably in formulations that had not been properly stabilized [28]. Drug stability is one of the key segments in quality assurance, and knowledge about the stability of the active ingredients is crucial for formulating suitable finished products [29]. Despite its frequent use, only a few studies have researched elementary vitamin D3 stability. It is known that various factors, such as exposure to light, humidity, oxygen, temperature, and pH may affect the stability of vitamin D3; however, the literature [30,31,32,33,34,35,36,37,38,39,40,41,42,43,44] provides qualitative and often contradictory data. As none of the published studies provides detailed data on its stability (e.g., pH profile, Arrhenius relationship), the focus of this study was to systematically and statistically evaluate vitamin D3 stability in aqueous solutions. Stability data was kinetically evaluated, which is an additional contribution of this study since vitamin D3 degradation kinetics have not yet been described. The effects of various factors (medium, light, temperature, oxygen, pH, and metal ions) on its stability were thus examined. The stability evaluation also included liquid finished products under three storage conditions. Additionally, potential approaches for improving vitamin D3 stability in aqueous solutions are proposed.

## 2. Materials and Methods

### 2.1. Materials

Vitamin D3 (98%), ascorbic acid (A), sodium hydroxide (NaOH), propylene glycol (PG), isopropanol (Isoprop) as well as acetonitrile (ACN), and methanol (MeOH) (both HPLC-grade) were purchased from Sigma-Aldrich (Steinheim, Germany). Hydrochloric acid (HCl, 37%), phosphoric acid (H_3_PO_4_, 85%), ethylenediaminetetraacetic acid disodium salt dihydrate (EDTA), citric acid (C), copper(II) chloride dehydrate (Cu^2+^), copper(I) iodide (Cu^+^), iron(II) sulfate heptahydrate (Fe^2+^), potassium dihydrogen phosphate (KH_2_PO_4_), and disodium hydrogen phosphate (Na_2_HPO_4_) were purchased from Merck (Darmstadt, Germany). A 30% H_2_O_2_ solution was purchased from Honeywell Fluka^TM^ (Seelze, Germany). Ethanol (EtOH, 96.6%) was purchased from Gram mol (Zagreb, Croatia). Oxygen (O_2_, 99.9%) was obtained from Messer, Slovenia. High purity Milli-Q water (MQ) was obtained through a Milli-Q A10 Advantage water purification system (Millipore Corporation, Bedford, MA, USA). Distilled water (DW) was obtained through an Elix^®^ Water Purification System (Merck, Darmstadt, Germany). Tap water (TW) was obtained from the laboratory at the Faculty of Pharmacy, University of Ljubljana (Ljubljana, Slovenia).

### 2.2. Instrumentation and Chromatographic Conditions

Vitamin D3 content was analysed according to a published stability-indicating method using a high-performance liquid chromatograph (HPLC) Agilent 1100/1200 Series instrument (Agilent Technologies, Santa Clara, CA, USA) equipped with a UV detector and ChemStation data acquisition system. In brief, a reversed-phase column Gemini C18 100 × 3.0 mm, 3 µm particle size column (Phenomenex, Torrance, CA, USA) at 40 °C was used with a mobile phase of acetonitrile water (99:1, *v/v*) at a flow rate of 1 mL/min. The detection was carried out at 265 nm [42]. The injection volume for samples within the vitamin D3 stability study in aqueous solutions was 20 µL, except for the samples with different vitamin D3 concentrations (between 2 and 50 µL). The injection volume for analysis of the tested finished products was adjusted to vitamin D3 content (between 2 and 20 µL).

### 2.3. Sample Preparation

#### 2.3.1. Samples for Vitamin D3 Stability Study in Aqueous Solutions

A fresh stock vitamin D3 solution (1000 mg/L) was prepared daily by precisely dissolving 10 mg of vitamin D3 standard in a 10.0 mL flask with MeOH. Further dilutions of this solution were prepared in various solvents to a concentration of 20 mg/L to evaluate the effect of various factors on vitamin D3 stability (Table 1). An additional stock solution with 5000 mg/L of vitamin D3 in MeOH was prepared and properly diluted with MQ and MeOH to evaluate the concentration effect in solutions containing 10% MeOH in MQ. To evaluate the effect of temperature, the solutions were stored at 25 and 40 °C in a VC 4034 climatic chamber (Vötsch, Reiskirchen-Lindenstruth, Germany) and ICH 260L climatic chamber (Memmert, Schwabach, Germany), respectively, and at 4 °C in a refrigerator (Gorenje, Velenje, Slovenija). Buffer solutions with different pH values were prepared by mixing appropriate volumes of 50.0 mM KH_2_PO_4_ and Na_2_HPO_4_ solutions in DW to the same volume (50 mL). Their pH values were measured using a calibrated pH meter and adjusted to the desired value with dilute H_3_PO_4_ or NaOH. Aqueous solutions were exposed to daylight in clear vials for up to 6 h to evaluate the effect of light. The effect of oxygen was evaluated by comparing aqueous solutions blown with oxygen every few hours with the control group (same concentration and storage conditions subjected to air). Additionally, different volumes of the aqueous solutions were transferred into vials to evaluate the impact of the air space. The effects of metal ions (Fe^2+^, Cu^+,^ and Cu^2+^) were evaluated by dissolving FeSO_4_ ∙ 7H_2_O, CuI, or CuCl_2_ ∙ 2H_2_O in MQ, in which the stock solution was diluted. Vitamin D3 stability was also evaluated in the presence of a combination of Cu^2+^ ions (2 mM) and different concentrations of ascorbic acid. The effects of various EDTA, ascorbic acid, and citric acid concentrations were also evaluated. Antioxidant stabilization efficiency was further evaluated in their combinations (more data is provided in Appendix A Table A1).

Each sample was prepared in at least three replicates. All samples were stored in amber vials, except for those used for studying the influence of light, and at 25 °C, apart from the samples used to examine the influence of oxygen (15 °C) and temperature (4, 25, and 40 °C). The vitamin D3 concentration was 20 mg/L in all samples, except when evaluating the effect of its concentration.

#### 2.3.2. Vitamin D3 Stability Study in Liquid Commercial Products

Three different medicines and six food supplements (FSs), commercially available on the European market, all in the form of oral drops were analysed. The analysed products included: AD3 (Krka, Novo Mesto, Slovenia), Baby-D (Jamieson, Toronto, ON, Canada), D-drops (Calivita International, California City, CA, USA), D-vitamin Olja (DunMedic, Stockholm, Sweden), Plivit D3 (Pliva, Zagreb, Croatia), Liquid vitamin D3 (Nutrilab, Irvine, CA, USA), Vigantol (Merck, Darmstadt, Germany), Vitamin AD3 drops (Iva-Farm, Valjevo, Serbia) and Vitamin D droplets (Jamieson, Toronto, ON, Canada). More detailed information on the analysed products is summarized in Table 2. After opening, every sample (prepared in triplicate) was directly injected into a high-performance, liquid-chromatography system with ultraviolet detection (HPLC–UV) to obtain the initial vitamin D3 content. The products were further stored in climatic chambers at 25 and 40 °C, and in a refrigerator at 4 °C. The samples were analysed regularly within six months to assess vitamin D3 stability. The results of the vitamin D3 stability study in the finished products are presented as a percentage of its content in relation to the initial content along with the standard error of the mean.

### 2.4. Kinetics Calculations and Data Analysis

The results are expressed as the mean of at least three different measurements along with the standard error of the mean. Zero-, first- and second-order kinetics as well as the Weibull model were fitted to vitamin D3 degradation by using a nonlinear least-square regression function from base “stats” package in R statistical software [45]. Fitted models were evaluated by R-squared and the Akaike information criterion (AIC). The rate constants for different stability conditions were determined based on the most adequate model. If comparable to Weibull kinetic model, the first-order model was primarily used due to its simplicity. The estimated rate constants were analysed with one-way ANOVA and further compared by 95% confidence intervals and post hoc tests (Tukey and Bonferroni-Holm post hoc test). MS Excel (Excel version 2013) and R (R version 4.0.2) software were used for data analysis.

## 3. Results

### 3.1. Degradation Kinetics

The modelling process included the experimental data obtained from the vitamin D3 stability study in aqueous solutions. The fitting of several kinetic models to vitamin D3 degradation is shown under the influence of light, oxygen, and other tested conditions (representative data provided for distilled water, Cu^2+^ ions, and pH = 1) (Table 3). The model with the highest coefficient of determination (*R*^2^) and the lowest Akaike information criterion (AIC) was selected.

The degradation of vitamin D3 in almost all cases followed the first-order kinetic model (Table 3—other conditions). The first-order kinetic model was less suitable for its degradation under exposure to light and oxygen. In these two cases, vitamin D3 degradation was most adequately described by the Weibull model. For vitamin D3 degradation in other tested conditions, Weibull kinetic model was found comparable to the first-order model, but the latter was used due to its simplicity.

### 3.2. Vitamin D3 Stability Study in Aqueous Solutions

The influence of various factors including temperature, pH, concentration, light, oxygen, and metal ions on the stability of vitamin D3 in aqueous solutions was systematically investigated and quantitatively and statistically evaluated.

#### 3.2.1. Effect of the Media

**Figure 1:**
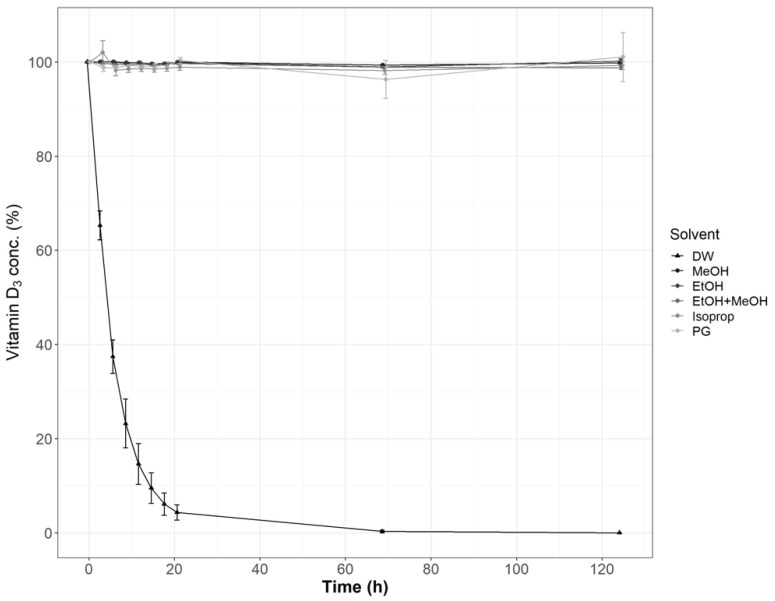
Effect of the media on vitamin D3 stability at 25 °C (*n* = 3).

Vitamin D3 stability was evaluated in DW and selected organic solvents including EtOH, MeOH, their mixture (50:50, *v/v*), Isoprop, and PG. Contrary to aqueous solutions, vitamin D3 was very stable in the tested non-aqueous solutions within the tested period of five days (Figure 1). Differences among the non-aqueous solutions were not significant (ANOVA, *p* > 0.05). Vitamin D3 was found very unstable in DW as its concentration dropped below 10% of the initial content after the first day of storage at 25 °C (Figure 1). Its stability was also significantly different in the three evaluated aqueous media: MQ, DW, and TW. Vitamin D3 was found to be the most stable in MQ and the least stable in DW (Table 4).

#### 3.2.2. Temperature Effect

Vitamin D3 stability was evaluated at three isothermal conditions: 4, 25 and 40 °C in three different aqueous media: MQ, DW, and TW. The results of the impact of temperature on vitamin D3 stability are presented by the first-order rate constants (k_1_) in Table 4. Higher temperature led to a statistically significant increase in reaction rate constants. High correlations (*R*^2^) between calculated rate constants and temperature showed that vitamin D3 degradation in all three aqueous media followed the Arrhenius equation, based on which activation energies were calculated.

#### 3.2.3. pH Effect

**Figure 2:**
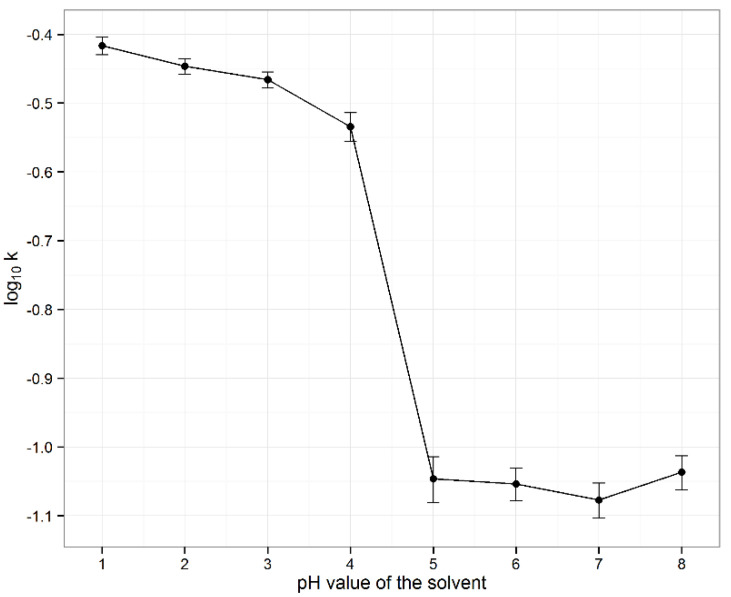
pH profile of vitamin D3 in aqueous solutions at 25 °C (*n* = 3).

The impact of pH in the range between 1 and 8 at 25 °C on the stability of vitamin D3 was systematically evaluated. First-order rate constants fitted vitamin D3 degradation well and were calculated for each pH value based on the time points up to the 6th hour (Figure 2). With a steep drop in stability between pH values 4 and 5, vitamin D3 was found most stable at a pH above 5. Statistical method ANOVA revealed no significant differences between the first-order rate constants at pH values between 5 and 8 (*p* ˃ 0.05). Vitamin D3 was found very unstable under acidic conditions, with an apparent trend of decreasing stability towards lower pH values, with statistically significant differences in the first-order rate constants at pH values between 1 and 4, as showed by ANOVA (*p* < 0.05) and Bonferroni-Holm post hoc test (*p* < critical values).

#### 3.2.4. Effect of Vitamin D3 Concentration

The effect of different vitamin D3 concentrations on its stability in aqueous solutions was further evaluated. Vitamin D3 stability was found concentration-dependent, with significantly higher first-order rate constants in solutions with its lower concentrations (Table 5).

#### 3.2.5. Effect of Light Exposure

**Figure 3:**
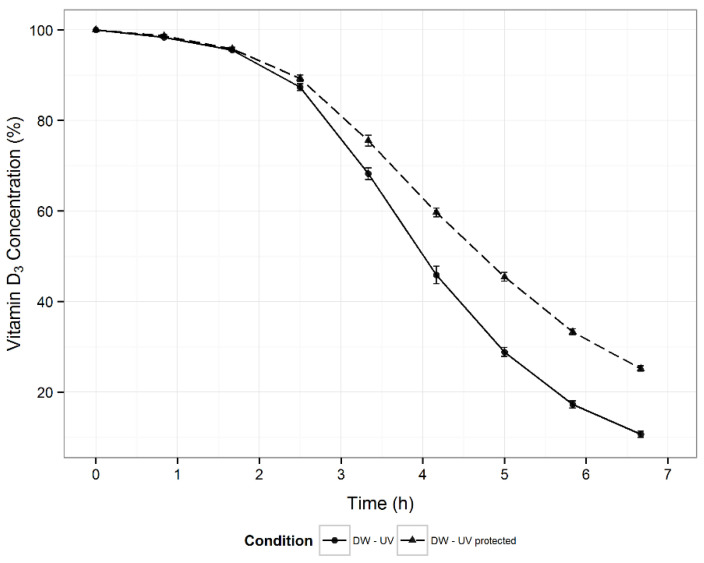
Stability of vitamin D3 in distilled water solutions exposed to light (DW–UV) and protected from light (DW–UV protected) at 25 °C (*n* = 5).

The effect of light on vitamin D3 stability was also evaluated. Its content in DW was higher at all time-points in samples protected from light (Figure 3). Based on the parameters of the fitted Weibull model, the differences in degradation kinetics were statistically significant. Presence of light reduced the scale parameter (λ) by 1.04 h (*p* < 0.001) and increased the shape parameter (*k*) by 0.36 units (*p* < 0.001).

#### 3.2.6. Effect of Oxygen

**Figure 4:**
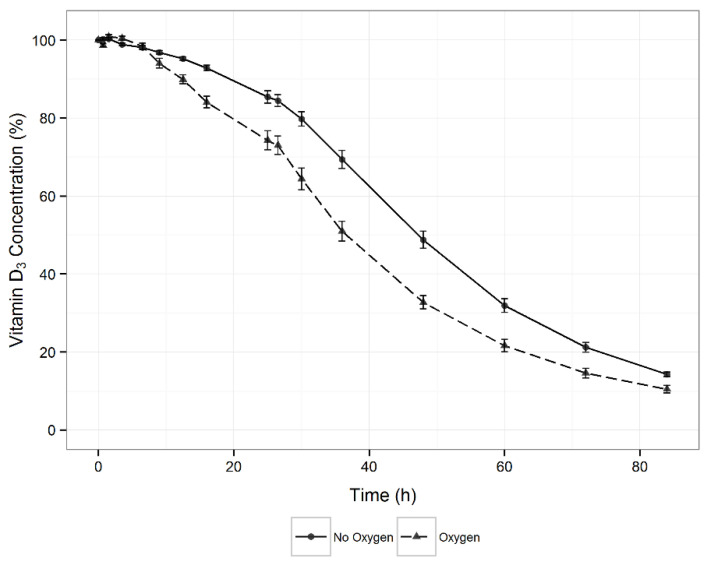
Effect of oxygen on vitamin D3 stability at 15 °C (*n* = 5).

To clarify whether exposure to oxygen affects the stability of vitamin D3, an experiment was conducted in which its aqueous solution was blown with oxygen. The results (Figure 4) were compared with the control samples using the Weibull model, which most adequately described vitamin D3 degradation under these conditions. The fitted model showed a statistically significant difference in vitamin D3 degradation, as the presence of oxygen reduced the scale parameter (λ) by 13.7 h (*p* < 0.001) and shape parameter (*k*) by 0.44 units (*p* < 0.001) of the Weibull model.

An additional experiment was conducted to evaluate whether the air space above the solution (headspace in vials) affects vitamin D3 stability. The samples were prepared in a mixture of MeOH and DW (75:25, *v/v*), as a medium in which vitamin D3 is more stable and permits a more accurate evaluation of its degradation. Comparing the degradation rates of vitamin D3 in 2 mL vials with different amounts of the same solution (from 0.5 to 2.0 mL, Table 1) it was evident that the air above the solution did not significantly affect its stability (ANOVA, *p* ˃ 0.05).

#### 3.2.7. Effect of Metal Ions

The effects of different concentrations of Fe^2+^, Cu^+^, and Cu^2+^ ions, as oxidation catalysts, in vitamin D3 aqueous solution were evaluated (Table 1). Metal ions-mediated vitamin D3 degradation occurred very quickly, regardless of the particular metal ion and its concentration. Nevertheless, Fe^2+^ ions were found most efficient, since even the lowest tested concentration of Fe^2+^ ions (0.01 mM) caused complete vitamin D3 degradation in less than two hours. The catalytic activity of Cu^+^ and Cu^2+^ ions on the degradation of vitamin D3 was comparable at all three tested concentrations, leading to complete degradation in approximately six hours. However, Cu^+^-mediated degradation of vitamin D3 showed greater variability than Cu^2+^-mediated degradation.

To investigate vitamin D3 degradation in more detail, the effects of a mixture of Cu^2+^ ions and different concentrations of ascorbic acid were evaluated. Vitamin D3 solution without the addition of Cu^2+^ ions and ascorbic acid was the control sample. Similarly as previously noted, Cu^2+^ (2 mM) caused very fast vitamin D3 degradation (significantly higher first-order rate constants compared to the control sample, Table 6). The expected the prooxidative effect of the combination of ascorbic acid and Cu^2+^ ions was not observed. Contrary, the antioxidant activity of ascorbic acid was observed in all solutions, except in the solution with the lowest ascorbic acid concentration (insignificant difference from the solution with the same Cu^2+^ concentration, without ascorbic acid). Ascorbic acid at a concentration ≥ 500 mg/L eliminated the degradation effect of the copper ions. The highest tested ascorbic acid concentrations (1000 and 2000 mg/L) additionally stabilized the vitamin D3 aqueous solution, as the degradation rate of these two solutions was significantly lower than in the control sample (Table 6).

### 3.3. Approaches towards Vitamin D3 Stabilization in Aqueous Solutions

Approaches towards stabilizing vitamin D3 in an aqueous solution by the addition of EDTA, citric acid, and ascorbic acid were undertaken. Their stabilizing capability, individually and in various combinations was determined and quantitatively evaluated. The results were statistically analysed by ANOVA and a Tukey post hoc test.

#### 3.3.1. Addition of an Individual Antioxidant

**Figure 5:**
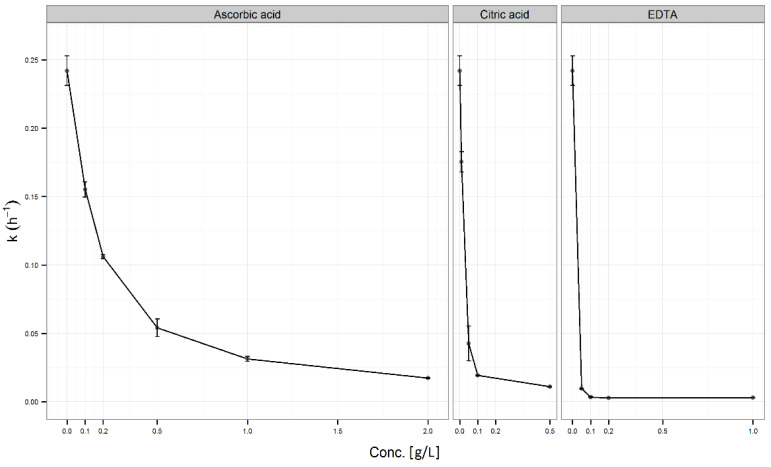
First-order rate constants for vitamin D3 degradation as a function of different concentrations of ascorbic, citric acid, or EDTA at 25 °C (*n* = 3).

The results of the effect of various concentrations of each antioxidant (EDTA, ascorbic or citric acid) on vitamin D3 stability are presented in Figure 5. The antioxidant activity of ascorbic acid was observed at all tested concentration levels. First-order rate constants in the presence of ascorbic acid were significantly lower (*p* ˂ 0.001) than in the control sample (without the addition of ascorbic acid). The stabilization effect of ascorbic acid significantly increased with its concentration (*p* ˂ 0.001). At the maximum-tested concentration (2.0 g/L), where the reaction rate was about 10-fold lower than in the control sample, ascorbic acid was unlikely to have achieved the maximum effect. 

The statistically significant stabilisation of citric acid was observed at all tested concentration levels compared to the control sample (Figure 5). The curve of rate constants as a function of antioxidant concentration for citric acid was similar to that of ascorbic acid, though the maximum citric acid concentration (0.5 g/L) stabilized vitamin D3 significantly better than the maximum ascorbic acid concentration (*p* < 0.001).

**Figure 6:**
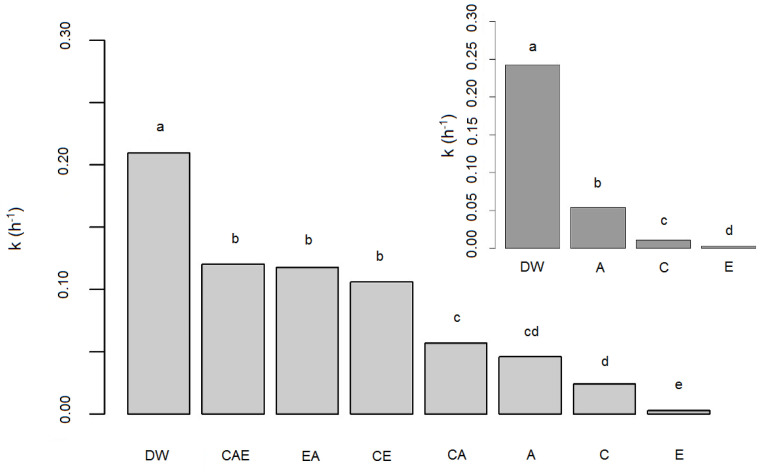
Vitamin D3 stabilization, presented by its first-order rate constants at 25 °C in distilled water (DW) and after the addition of an individual antioxidant EDTA (E), citric acid (C), ascorbic acid (A), and combinations of two and all three antioxidants (CA, CE, EA, and CAE) (*n* = 3). The columns designated by different letters showed statistical differences from the other groups (*p* < 0.001). Insert—The effect of the same concentration (0.5 g/L) of citric acid (C), ascorbic acid (A), or EDTA (E) on vitamin D3 degradation rate at 25 °C (*n* = 3). The columns designated by different group letters showed statistical differences from other groups (*p* < 0.001).

EDTA was found to be the most effective among the selected antioxidants in the stabilization of vitamin D3 solutions (Figure 5), which was statistically confirmed. The lowest tested EDTA concentration (0.05 g/L) stabilized vitamin D3 better than the highest tested ascorbic acid concentration (*p* < 0.001). EDTA reached a maximum stabilizing effect at a concentration of 0.1 g/L (no significant differences between the tested EDTA concentrations above 0.1 g/L, *p* ˃ 0.05). Statistical evaluation of individual antioxidants (EDTA, ascorbic, and citric acid) at the same concentration (0.5 g/L) showed that EDTA stabilized vitamin D3 statistically significantly better than ascorbic or citric acid (Figure 6-Insert). It is also evident that 0.5 g/L of citric acid statistically significantly decreased vitamin D3 degradation compared to DW or solution containing 0.5 g/L of ascorbic acid.

#### 3.3.2. Addition of Antioxidant Combinations

Samples with combinations of these three antioxidants—EDTA, ascorbic, and citric acid––in various concentrations were prepared and evaluated to find a potential synergism among antioxidants. The statistical evaluation of the antioxidant’s effectiveness for vitamin D3 stabilization included combinations of two and three antioxidants, as well as individual antioxidants as described in Table 1 and Appendix A Table A1. Statistical method ANOVA revealed differences between the groups (*p* < 0.001). The Tukey post hoc test with respect to the heterogeneity of variances between groups showed statistical differences among groups as designated by different letters in Figure 6. The addition of antioxidants (individually or in combination) significantly stabilized vitamin D3 compared to its stability in DW (column a). However, EDTA added individually stabilized vitamin D3 aqueous solution more significantly than in any tested combination (column e). Among the tested antioxidant combinations, the combination of citric and ascorbic acid (CA) with a known synergistic effect [46] (column c) was found most effective, while the combination of all three antioxidants (column b) was least effective. In view of these unexpected results, the same experiment was repeated and comparable results were obtained.

### 3.4. Vitamin D3 Stability in Liquid Commercial Products

A study on three medicines and six FSs, all in the form of oral drops and containing vitamin D3 as the main active ingredient, was also performed to evaluate its stability in various formulations. Vitamin D3 stability in these products was evaluated at three isothermal conditions: 4, 25, and 40 °C. The declines in vitamin D3 content were evaluated by first-order kinetics, which fitted its degradation well. Its content remained unchanged after 6 months of storage at ambient temperature in approximately half of the tested products and gradually decreased in the other half by up to almost 100% (Table 7).

Regarding ambient storage conditions, vitamin D3 was found to be properly stabilized in all tested oil-based formulations with <10% degradation after 6 months. However, the observed degradation after 6-months of storage at an elevated temperature implied that the oil type and its quality is also an important factor for vitamin D3 stability. Vitamin D3 was generally less stable in the water-based formulations (Table 7).

The temperature dependence of vitamin D3 stability was also observed within the finished products’ stability study. The effect of the storage temperature on vitamin D3 stability is most evident in products 3, 6, 7, and 9 (Table 7). Vitamin D3 degraded most significantly in FS 5, 7, and 9. The higher storage temperature caused a significant increase in vitamin D3 first-order rate constants in all three water-based FSs. Similar to the three different tested aqueous media (TW, DW, and MQ), vitamin D3 degradation in these three finished products also followed the Arrhenius equation (high coefficient of determination *R*^2^). The calculated activation energy for vitamin D3 in Product 5 correlated well with the activation energies in TW, while in Products 7 and 9 it correlated with DW (Table 4 and Table 7).

The performed stability study on vitamin D3 in finished products also provided valuable information on their shelf life after opening at which time medicines contain >90% and FSs >80% of the vitamins claimed on the label [47]. After being open for at least 6 months, shelf life was confirmed for products 1, 2, 3, 4, 6, and 8 based on the results of the long-term stability study (Table 7). The calculated shelf life after opening for products 5, 7, and 9 were approximately 11 days, 5 months, and 44 days, respectively, but these could be significantly extended to ˃3 months for Product 5, ˃22 months for Product 7, and ≈5 months for Product 9 if they were stored at a lower temperature (refrigerator).

## 4. Discussion

The main aim of this study was to establish the stability of vitamin D3 in a comprehensive and quantitative manner, which had not yet been described in the literature. The first step was to determine the vitamin D3 degradation kinetics. The selection of the most appropriate kinetic model was based on two statistical parameters *R*^2^ and AIC, since linear regression may overestimate or underestimate the reaction rate depending on the deviation of the experimental data from linearity [48]. The first-order kinetic model was suitable for vitamin D3 degradation under all tested conditions, except light and oxygen, probably due to more complex reaction mechanisms. In these two cases, the established models in chemical kinetics were found to be inappropriate. Therefore, the Weibull kinetic model was introduced, which included a scale constant in addition to the rate constant, thus adding flexibility and well-described vitamin D3 degradation under all tested conditions. In systems with simpler vitamin D3 degradation (all tested conditions except light and oxygen), it was comparable to the first-order model, which was used primarily for its simplicity. The Weibull model has lately found useful application in fields such as modelling microbial reactions [49,50,51], survival analysis [52,53,54], and engineering and materials [55,56,57,58], but it has rarely been applied to stability data [59].

The vitamin D3 stability study in aqueous solutions (to evaluate the influence of media, temperature, pH, concentration, light, oxygen, and metal ions on its stability) provided useful information for the formulation of stable vitamin D3 products. It also provided a significant upgrade of the existing knowledge on vitamin D3 stability, which is rather inconclusive and only qualitative. Moreover, this study is beneficial for the end-users to distinguish quality products among the wide variety of vitamin D3 products on the market.

The initial conclusion from the performed stability study is that vitamin D3 is very unstable in aqueous media. The significantly higher vitamin D3 stability observed in organic solvents (Figure 1) is probably due to the absence of hydronium and hydroxyl ions, used in hydrolysis, and catalysts for oxidation reactions, such as metal ions [60]. Among the three different tested waters (MQ, DW, and TW), vitamin D3 was found the most stable in MQ and the least stable in DW. Because of the unexpected results, this experiment was repeated and extended to three different temperatures: 4, 25, and 40 °C to confirm the trend (Table 4). Although temperature mostly accelerates chemical reactions, its role in oxidation reactions is composed as it may also act in the opposite direction due to the lower oxygen solubility at higher temperatures [61]. Therefore, the impact of temperature on compounds susceptible to oxidation, like vitamin D3, requires experimental evaluation. The obtained results followed the general principle: higher temperature led to a statistically significant increase in reaction rate constants (Table 4) and decreased vitamin D3 stability. High correlations (*R*^2^) between calculated rate constants and temperature showed that vitamin D3 degradation in all three aqueous media followed the Arrhenius equation. Obtained activation energies (Table 4) have not yet been published in the literature. Significantly different activation energies indicate alterations in vitamin D3 degradation mechanism in the particular aqueous media, which may be a consequence of metal ions, acting as catalysts in oxidation reactions. Metal ions, which are less likely to be present in ultrapure MQ, lower the activation energy and lead to faster vitamin D3 degradation (higher rate constant) in DW and TW. The results in Table 4 led to the following conclusion of practical importance: vitamin D3 in aqueous solutions may be stabilized by being stored at a lower temperature because there was a statistically significant decrease of first-order rate constants at a lower temperature. The stabilizing effect of lower storage temperature is stronger in MQ than in TW or DW.

Vitamin D3 stability in aqueous solutions was also evaluated for its concentration and the pH of the solutions as they are both important factors in chemical reactions in solutions. Since the pH profile for vitamin D3 has not yet been published, the impact of pH in the range between 1 and 8 was systematically evaluated at 25 °C. Vitamin D3 was found unstable in acidic media and more stable at pH values above 5 (Figure 2). These results were in contrast to the usual trend for oxidation: lower stability at higher pH [37]. We consider that the first report of a pH profile for vitamin D3 provided beneficial information about the existing qualitative knowledge on its stability in neutral and weak alkaline [38,39] and instability in acidic media [39,43]. By evaluating the concentration effect, we concluded that aqueous solutions with lower vitamin D3 concentrations were less stable (Table 5) as a consequence of the greater impact of particular destabilizing factors.

Vitamin D3 degradation is mostly mediated through oxidative reactions [34,59,60,61]. Factors that could induce and influence oxidation (e.g., light, oxygen, and metal ions) were thus evaluated. Light induces oxidative reactions, mostly through the formation of free radicals, and causes isomerization. The obtained results of decreased vitamin D3 stability under the influence of light (Figure 3) are supported by several reports in the literature [31,36,38,39,40,41] although contradictory data are also found in the published literature [43,44]. Possible reasons for the conflicting data are different experimental conditions (e.g., exposure time, light sources and illumination, media, or concentration) or deficient information on the experimental conditions. In a recently published study on vitamin D3-forced degradation, its isomers were observed as degradation products under the influence of light [42], thus confirming the destabilizing effect of light on vitamin D3. Oxygen was also identified as a factor, which significantly decreased the stability of vitamin D3 (Figure 4). However, the air above the solution was not a determining factor for vitamin D3 destabilization.

Metal ions are widespread in the environment and may also be present in finished products. Their source may be the manufacturing equipment, containers, and the used water, or they may be present as impurities in the active ingredients or excipients if used as catalysts in the synthesis processes [62]. Trace metals can act as catalysts in oxidation processes and cause active ingredient degradation [63]. Vitamin D3 incompatibility with trace metals has been indicated in the literature but not experimentally supported [64]. In view of the importance of this subject and the lack of published data, experiments with Fe^2+^, Cu^+^, and Cu^2+^ ions were performed, which led to the conclusion that vitamin D3 degradation may be catalysed by different metal ions (Fe^2+^, Cu^+,^ and Cu^2+^). The potential prooxidative effect of the combination of ascorbic acid and Cu^2+^ ions, mediated through oxidation-reductive reactions and the formation of reactive species (hydrogen peroxide, hydroxyl, and ascorbic radicals) [65,66], was further evaluated. In this experiment, we only observed the antioxidant activity of ascorbic acid. These results indicate that ascorbic acid and other similar antioxidants could be used as stabilizers of aqueous vitamin D3 solutions, which was further investigated.

The performed stability study in aqueous solutions indicated that vitamin D3 could be stabilized by limiting the effects of destabilizing factors (e.g., storage at a lower temperature and/or at pH > 5) and active intervention by the addition of stabilizers, which was quantitatively evaluated. The addition of three well-known antioxidants: EDTA, citric acid, and ascorbic acid significantly stabilized vitamin D3 (Figure 5). The antioxidant activity of ascorbic acid is mediated through the reduction of reactive oxygen or nitrogen species or acts as a weak chelating agent [67,68]. Citric acid and EDTA effectively remove metal ions by chelation [67,68]. Evaluating their stabilization capability at the same concentration, significant differences in the stabilizing effect of EDTA, citric, and ascorbic acid were observed. The stabilizing effect decreased in the stated antioxidants order (Figure 6). Their stabilizing effect was concentration-dependent, except for EDTA at concentrations ≥0.1 g/L (Figure 5). The wide concentration range of ascorbic acid was selected in view of its potential prooxidative effect, which was not observed (Table 6). Based on the results, we concluded that the use of antioxidants such as EDTA, ascorbic acid, and citric acid, one of the basic techniques for preventing oxidation [68,69], is also beneficial for vitamin D3 stabilization.

Considering the results for vitamin D3 stabilization with individual antioxidants, we anticipated that a combination of two or three would further increase stability. The statistical evaluation, which included all obtained results on vitamin D3 stabilization by the addition of individual antioxidants and their combinations revealed the superior stabilizing effect of EDTA. The observed lower stabilization capability of the combinations may have arisen from the multifunctional effects of the individual stabilizers acting as antioxidants, prooxidants, chelating agents, reducing agents, and oxygen removers. Antioxidant interactions can result in alteration of the microenvironment of one antioxidant by another and competitive oxidation and regeneration between antioxidants [70]. The overall effect of their combination on the oxidation of other compounds is not entirely predictable, as also the catalysing power of iron ions is greater when they are in the complex of EDTA. Thus conducted and evaluated experiments are beneficial in view of the wide range of antioxidant concentrations, detection of the potential interactions, and illustration of real situations. Based on these results, we concluded that chelation and elimination of trace metals are crucial for vitamin D3 stabilization.

Basic studies and knowledge of vitamin D3 stability are necessary to ensuring its stability within finished products. Therefore, understanding its instability and acquiring expertise on stabilization approaches are required for providing quality, safe and efficient products with an appropriate shelf life. The knowledge, gained within the vitamin D3 stability study in aqueous solutions was further transferred to commercially available finished products. The results from the vitamin D3 stability study in three medicines and six FSs, all in the form of oral drops (Table 7), are in accordance with results from the stability study of vitamin D3 in solutions (Section 3.2 *Vitamin D3 stability study in aqueous solutions* and Section 3.3 *Approaches towards vitamin D3 stabilization in aqueous solutions*). Analogously as observed within the stability study in solution (Section 3.2.1 *Effect of the media*), we identified the carrier of the formulation as the main contributing factor for vitamin D3 stabilization. Contrary to the tested oil-based formulations, vitamin D3 was found to be unstable in water-based formulations, which are commonly found on the market. Among the tested water-based formulation, we observed a difference between vitamin D3 stabilization within the two tested product categories: medicines and FSs. Although unstable in aqueous solutions, vitamin D3 may be properly stabilized, which was achieved with the formulation of Products 2 and 3, both registered as medicines. The observed vitamin D3 instability in all three water-based FSs (5, 9, and 7) also confirmed our thesis that aqueous vitamin D3 solutions require proper stabilization, which had been neglected or deemed to be of lesser importance for their manufacturers. Namely, Product 7 did not contain any stabilizer or organic solvents. Vitamin D3 was the least stable in FS Product 5, leading to its total degradation after 6 months of storage at the recommended conditions (ambient temperature). Such pronounced vitamin D3 instability was in accordance with our findings, considering the acidic environment of this product (pH ˂ 3), which was identified as one of the key destabilizing factors within the vitamin D3 stability study in aqueous solutions (Section 3.2.3 *pH effect*). In comparison, the other tested water-based formulations were in the more favourable pH range according to its pH profile (between 5.6 and 7.2). Also, the composition of Product 5 did not provide stabilization because it is an aqueous vitamin D3 solution containing only one potential vitamin D3 stabilizer—citric acid. However, its presence at high concentrations had a destabilizing effect on vitamin D3 because it created an acidic environment.

The findings, presented in Section 3.3 *Approaches towards vitamin D3 stabilization in aqueous solutions* indicated the importance of the products’ excipients as they may contribute towards vitamin D3 stabilization. Product 2 (Table 7) is an example of the proper stabilization of aqueous vitamin D3 solutions, which was achieved by adding butylated hydroxytoluene and citric acid as antioxidants. Product 3 was the least stable tested medicine. However, with 4% degradation after 6 months of storage at room temperature, it was an acceptable formulation for vitamin D3 stability. Vitamin D3 stabilization in this product was most probably achieved by the addition of organic solvents (propylene glycol and ethanol), in which vitamin D3 was found to be quite stable (Section 3.2.1 *Effect of the media*). Different vitamin D3 stabilization in the tested products was also reflected in the different shelf life provided by the manufacturers (between 1 and 5 years). Only one of the tested products, Product 1, specified a shelf life after opening of 6 months, which was confirmed in this study.

The determined temperature dependence of vitamin D3 stability in aqueous solutions (Table 4) was also confirmed by the comparable activation energies in the less stable products (Table 7). An interesting and practical conclusion, based on temperature dependence, is the possibility of a significant extension of shelf life after opening by storing the products at a lower temperature. On the other hand, considering the high stability of vitamin D in Product 2 (Table 7), refrigerator storage, as recommended by the manufacturers, seems unnecessary. These results also provide a foundation to redefine the recommended storage conditions.

Vitamin D3 stability in aqueous solutions was found to be concentration-dependent (Table 5). Considering these results, vitamin D3 concentration presumably also contributes to its stability in finished products. However, despite having high stability in the medicine and FS with the highest vitamin D3 concentration tested products, a straightforward relation could not be postulated because of the different compositions of the tested products. Considering the different levels of stability in products with a comparable vitamin D3 concentration (Products 2 and 7 and Products 3 and 8, Table 7) and the high concentration in the least stable product (FS (5) in Table 7), it can be concluded that vitamin D3 stability in finished products is primarily dependent on the formulation (medium and excipients). Vitamin D3 stability in solutions was also found dependent on its exposure to light (Section 3.2.5 *Effect of light exposure*). All manufacturers recognized the risk of vitamin D3 degradation under the influence of light and thus selected containers, which provide light protection (amber glass or plastic bottles).

The results on vitamin D3 stability in finished products were also compared to the results of a previous preliminary stability study [28], performed on different LOTs of the same three medicines (Products 1, 2, and 3 in Table 2). The stability of vitamin D3 in Products 1 and 2 was comparable. Although vitamin D3 was the least stable among the tested medicines in Product 3 (Table 7), its stability had been considerable improved within this product during the 2.5 years between both stability studies. Namely, the revealed ≈15% decline after 6 months at 25 °C and almost 50% decline at 40 °C within the preliminary study [28] was reduced to ≈4% at 25 °C and ≈20% decline at 40 °C (Table 7). Aware of the suboptimal vitamin D3 stability within the formulation, the manufacturer presumably aimed toward improving stabilization. Because the same qualitative compositions were on the labels of both tested LOTs, we assumed that the manufacturer changed the proportions between the excipients to achieve better vitamin D3 stability.

The tested antioxidants in Section 3.3 *Approaches towards vitamin D3 stabilization in aqueous solutions* (ascorbic acid, citric acid, and EDTA) are physiologically acceptable and suitable for stabilizing vitamin D3. Since EDTA was found to be the most efficient antioxidant in aqueous vitamin D3 solutions (Figure 6), adding it to increase vitamin D3 stability in finished products would be a valuable proposal.

## 5. Conclusions

Vitamin D3 degradation pathways and kinetics are rather complex and unclear. The effects of various factors (media, temperature, pH, concentration, light, oxygen, metal ions) on its stability in aqueous solutions were clarified in this study, which could help resolve the confusion and contradictions in the literature regarding its stability. The comprehensive and quantitative stability evaluation initially required the selection of the most appropriate model to describe the degradation kinetics. The established kinetic models (zero-, first- and second-order kinetics) did not fit well the degradation of vitamin D3 under all conditions, thus necessitating a more flexible and advanced approach. The Weibull model, which is an upgrade of the commonly used models, was found appropriate for degradation under all tested conditions, including the more complex degradation under the influence of light and oxygen. In cases other than these two, vitamin D3 degradation also followed first-order kinetics, which was applied because of its simplicity. Vitamin D3 was found to be very unstable in aqueous solutions and particularly sensitive to the presence of metal ions and low pH values. Nevertheless, liquid vitamin D3 products are commonly aqueous solutions, which requires appropriate stabilization. The results obtained from the vitamin D3 stability study in liquid products disclosed significantly lower vitamin D3 stability in water-based compared to oil-based formulations. We also concluded that manufacturers of food supplements pay less attention to stability even though it is essential for assuring quality, safety, and efficacy. The results obtained from the vitamin D3 stability study in aqueous solutions and commercial liquid products highlight the importance of a suitable formulation, mainly by the selection of proper pH value, elimination of metal ions, and addition of certain stabilizers. These results are undoubtedly a useful foundation for the formulation of stable vitamin D3 products given their frequent worldwide use.

## Figures and Tables

**Table 1:** Selected parameters along with tested conditions within the vitamin D3 stability study in aqueous solutions (vitamin D3 concentration = 20 mg/L unless otherwise stated).

Parameter	Media	Varied Condition
media	different	aqueous (DW, TW, MQ) and organic (EtOH, EtOH + MeOH, Isoprop., MeOH, PG)
temperature	TW/DW/MQ	4 °C, 25 °C, 40 °C
pH	DW	pH: 1, 2, 3, 4, 5, 6, 7, 8
concentration	MQ -MeOH (90:10, *v/v*)	10, 25, 100 and 500 mg/L
light	DW	clear vials exposed to daylight, amber vials kept in the dark
oxygen	DW	with or without O_2_
air space	MeOH–DW (75:25, *v/v*)	solution volume in 2-mL vials (0.5, 1.0, 1.5 and 2.0 mL)
Fe^2+^, Cu^+^ or Cu^2+^ ions	MQ	0.01, 0.05, 0.1 mM
Cu^2+^ + A	DW	CuCl_2_ (2 mM) + A (0, 100, 200, 500, 1000, 2000 mg/L)
A	DW	A (100, 200, 500, 1000, 2000 mg/L)
EDTA	DW	EDTA (50, 100, 200, 500, 1000 mg/L)
C	DW	C (5, 10, 50, 100, 500 mg/L)
A, EDTA and/or C *	DW	A (500, 2000, 5000 mg/L); EDTA (200, 500, 1000 mg/L); C (5, 50, 500 mg/L)

A—ascorbic acid; C—citric acid; DW—distilled water; EtOH—ethanol; EtOH + MeOH—50:50, *v/v*; Isoprop—isopropanol; MeOH—methanol; MQ—Milli-Q water; PG—propylene glycol; TW—tap water; * Combinations of two or three antioxidants in different concentrations (see Appendix A Table A1).

**Table 2:** Data on the tested products.

Product	Type	Vitamin D3 Content ^*^	Ingredients		
			Carrier	Potential Stabilizers	Other Excipients
1	M	20,000 IU/mL	MCT		
2	M	4000 IU/mL	PW	BHT, anhydrous citric acid, propylene glycol	methyl parahydroxybenzoate, MGHS, macrogol 400, anhydrous sodium hydrogen phosphate
3	M	2000 IU/mL	PW	glycerol, propylene glycol	MGHS, sodium benzoate, sucrose, sodium saccharinate, orange aroma (ethanol)
4	FS	1000 IU/drop	MCT		
5	FS	12,000 IU/mL	deionized water	citric acid, vegetable glycerine	natural orange aroma, lemon essential oil
6	FS	400 IU/drop	corn oil		
7	FS	4000 IU/mL	PW		sodium benzoate
8	FS	80 IU/drop	vegetable oil		
9	FS	320 IU/mL	PW	vegetable glycerine	stevia, natural cherries aroma, potassium sorbate, and sodium benzoate

BHT—butylated hydroxytoluene; FS—food supplement; M—medicine; MCT—medium-chain triglycerides; MGHS—macrogolglycerol hydroxystearate; PW—purified water. * as stated on the packaging of the product.

**Table 3:** Kinetic models for vitamin D3 degradation under different conditions.

	Light		Oxygen		Other Conditions *					
					DW		Cu^2+^		pH = 1	
Model	*R* ^2^	AIC	*R* ^2^	AIC	*R* ^2^	AIC	*R* ^2^	AIC	*R* ^2^	AIC
0. order: y = 100 − k × t	0.906	344.8	0.947	475.6	0.892	164.8	0.758	109.8	0.826	102.6
1. order: y = 100 ∗ exp(−k × t)	0.796	379.4	0.918	505.0	0.982	127.3	1.000	14.30	0.995	61.45
2. order: y = 100/(1 + k × t)	0.698	397.1	0.843	549.0	0.903	162.5	0.986	75.55	0.989	69.02
Weibull:y = 100 ∗ exp(−(t/λ)^*k*)	0.994	225.7	0.973	431.8	0.997	89.28	1.000	1.616	0.998	52.43

*R*^2^—coefficient of determination; AIC—the Akaike information criterion; k—slope (rate constant); t—time [h]; λ—scale parameter; *k*—shape parameter; * representative data provided for distilled water, Cu^2+^ ions, and pH = 1.

**Table 4:** First-order rate constants for vitamin D3 at various temperatures in Milli-Q (MQ), distilled (DW), and tap water (TW), along with the Arrhenius equation.

	DW	TW	MQ
k_1_ (4 °C) (h^−1]^ (CI)	0.027 (0.023–0.031)	0.012 (0.009–0.014)	0.001 (0.000–0.002)
k_1_ (25 °C) (h^−1^) (CI)	0.143 (0.124–0.162)	0.099 (0.096–0.102)	0.024 (0.021–0.026)
k_1_ (40 °C) (h^−1^) (CI)	0.400 (0.385–0.416)	0.339 (0.270–0.407)	0.085 (0.077–0.092)
Arrhenius equation	y = −6465x + 19.74	y = −8142x + 24.96	y = −10,725x + 31.97
*R* ^2^	1.000	0.999	0.988
Ea (kJ/mol)	53.8	67.7	89.2

CI—95% confidence interval; Ea—activation energy.

**Table 5:** First-order rate constants for vitamin D3 at different concentrations in Milli-Q water-methanol (90:10, *v/v*) at 25 °C (*n* = 3).

Vitamin D3 Concentration	10 mg/L	25 mg/L	100 mg/L	500 mg/L
k_1_ (h^−1^) (CI)	0.055 (0.050–0.060)	0.047 (0.046–0.049)	0.035 (0.032–0.037)	0.024 (0.024–0.025)

CI—95% confidence interval.

**Table 6:** First-order rate constants for vitamin D3 degradation in the presence of Cu^2+^ ions (alone – point 0) and in combination with different concentrations of ascorbic acid (A) at 25 °C (*n* = 3).

	A Conc. (mg/L)	k_1_ (h^−1^)	CI
	Control *	0.131	0.122–0.141
A + 2 mM Cu^2+^	0	0.526	0.505–0.548
	100	0.518	0.495–0.542
	200	0.267	0.246–0.291
	500	0.122	0.101–0.147
	1000	0.036	0.033–0.038
	2000	0.038	0.033–0.043

CI—95% confidence interval; * vitamin D3 in the same media without the addition of ascorbic acid and Cu^2+^ ions.

**Table 7:** Remaining vitamin D3 with respect to initial content in the tested liquid commercial products after 6 months of storage at 4, 25, and 40 °C (average ± relative standard error, *n* = 3).

Vitamin D3 Content (%) after 6 Months in Oil-Based Formulations				Vitamin D3 Content (%) after 6 months in Water-Based Formulations			
	**4 °C**	**25 °C**	**40 °C**		**4 °C**	**25 °C**	**40 °C**
**M (1)**	102.0 ± 0.2	101.0 ± 0.1	98.3 ± 0.3	**M (2)**	101.5 ± 0.0	101.9 ± 0.9	102.3 ± 0.0
**FS (4)**	101.2 ± 0.2	99.7 ± 0.2	97.5 ± 0.2	**M (3)**	96.7 ± 0.0	96.0 ± 1.0	80.5 ± 2.0
**FS (6)**	91.2 ± 2.4	89.1 ± 0.1	46.8 ± 0.4	**FS (5)**	66.2 ± 2.8	1.5 ± 0.9	0.0 ± 0.0
**FS (8)**	102.9 ± 1.0	102.2 ± 0.6	100.2 ± 0.5	**FS (7)**	93.1 ± 0.5	79.4 ± 1.0	40.6 ± 1.2
				**FS (9)**	76.6 ± 3.5	39.8 ± 2.6	2.3 ± 0.2
**Quantitative Evaluation of Vitamin D3 Stability in the Unstable Products** **(First-Order Constants and Arrh. Data)**							
	**k_1_ (4 °C)** **(month^−1^) (CI)**	**k_1_ (25 °C)** **(month^−1^) (CI)**		**k_1_ (40 °C)** **(month^−1^) (CI)**	**Arrh. eq.**	***R*^2^**	**Ea (kJ/mol)**
**FS (5)**	0.069 (0.057–0.081)	0.610 (0.362–0.859)		2.469 (2.141–2.797)	y = −8613x + 28.42	1.000	71.6
**FS (7)**	0.012 (0.010–0.013)	0.038 (0.035.042)		0.150 (0.142–0.159)	y = −5987x + 17.08	0.971	49.8
**FS (9)**	0.045 (0.032–0.058)	0.154 (0.136–0.173)		0.634 (0.605–0.662)	y = −6240x + 19.33	0.972	51.9

Arrh. eq.—Arrhenius equation; CI—95% confidence interval; Ea—activation energy; FS—food supplement; M—medicine.

## Data Availability

Data is contained within the article.

## Appendix A

**Table A1:** Information on the tested antioxidant combinations for vitamin D3 stabilization (vitamin D3 concentration was 20 mg/L in all cases).

Mark	C (mg/L)	EDTA (mg/L)	A (mg/L)
**CE 1**	5.00	50.0	-
**CA 1**	5.00	-	100
**EA 1**	-	50.0	100
**CEA 1**	5.00	50.0	100
**CE 2**	50.0	500	-
**CA 2**	50.0	-	500
**EA 2**	-	500	500
**CEA 2**	50.0	500	500
**CE 3**	500	200	-
**CA 3**	500	-	2000
**EA 3**	-	200	2000
**CEA 3**	500	200	2000
**CE 4**	500	1000	-
**CA 4**	500	-	5000
**EA 4**	-	1000	5000
**CEA 4**	500	1000	5000

CE—a combination of citric acid and EDTA; CA—a combination of citric and ascorbic acid; EA—a combination of EDTA and ascorbic acid; CEA—a combination of citric acid, EDTA, and ascorbic acid.
